# Validity of kinematics measures to assess handwriting development and disorders with a graphomotor task

**DOI:** 10.1192/j.eurpsy.2021.564

**Published:** 2021-08-13

**Authors:** C. Lopez, A. Cannafarina, L. Vaivre-Douret

**Affiliations:** 1 Faculty Of Society And Humanity, Division Psychology, Université de Paris, Paris, France; 2 National Institute Of Health And Medical Research (inserm Umr 1018-cesp), Faculty Of Medicine, University of Paris-Saclay, UVSQ, Villejuif, France; 3 Faculty Of Health, Division Of Medicine Paris Descartes, Université de Paris, Paris, France; 4 University Institute Of France, (Institut Universitaire de France, IUF), Paris, France; 5 Department Of Child Psychiatry, Assistance Publique-hôpitaux De Paris (ap-hp), Centre, Necker-Enfants Malades University Hospital, Paris, France; 6 Department Of Endocrinology, Imagine Institute, Necker-Enfants Malades University Hospital, Paris, France

**Keywords:** handwriting disorders, handwriting development, graphomotor task, Children

## Abstract

**Introduction:**

Handwriting disorder is considered to be one of the major public health problems among school-aged children worldwide. All the scales in the literature use handwriting tasks but it could be interesting to investigate a more accurate assessment of handwriting difficulties before the development and acquisition of handwriting as such.

**Objectives:**

The objective of our study is to examine the validity of a prescriptural task consisting of copying a line of cycloid loops in the diagnosis of handwriting disorders.

**Methods:**

35 children with handwriting disabilities and 331 typically developing right-handed children in primary school, aged 6-11 years old, were included in the study. They performed a copy of a line of cycloid loops, in an ecological setting, with a paper sheet put on the table. The kinematic measures were recorded with a digital pen. A Receiver Operating Characteristic method (ROC curve) was used to determine whether the loops line copy may be a sensitive test to diagnose handwriting disorders.

**Results:**

Six kinematic variables recorded during the prescriptural task were found to be relevant markers of handwriting disorders with a sensibility between 0.743 and 0.880: strokes number, total and effective drawing time, in-air pauses times, loops number, number of peaks velocity.

**Conclusions:**

The graphomotor task of copying a line of cycloid loops showed a good sensitivity to diagnose handwriting disorders and appeared to be a good predictor test, more particularly with the variables reflecting the strokes temporal organization.Drawing loops is a rapid graphomotor task, useful for exploring prerequisites of handwriting in screening for handwriting disorders.

